# Dynamic changes of lipid profile in severe hypertriglyceridemia-induced acute pancreatitis patients under double filtration plasmapheresis: a retrospective observational study

**DOI:** 10.1186/s12944-020-01383-8

**Published:** 2020-09-15

**Authors:** Huijun Zheng, Donghai Wang, Xiaoling Wang, Yongjun Lin, Zhihua Lu, Yueliang Chen, Guo Feng, Na Yang

**Affiliations:** Department of Critical Care Medicine, Sir Run Run Shaw Hospital, Zhejiang University School of Medicine, Hangzhou, China

**Keywords:** Severe hypertriglyceridemia-induced acute pancreatitis, Double filtration plasma apheresis, Lipid profile, High-density lipoprotein

## Abstract

**Background:**

To investigate the dynamic change of lipid profile under double filtration plasmapheresis (DFPP) in severe hypertriglyceridemia-induced acute pancreatitis (sHTGP) patients and ascertain the association between these changes and the clinical prognosis.

**Methods:**

sHTGP patients admitted within 72 h after disease onset were included, and all the patients received DFPP within 24 h after admission. Lipid profile were detected on admission, consecutive 4 days after DFPP and at discharge.

**Results:**

There were 47 sHTGP patients enrolled in this study. At admission, all the parameters of lipid profile changed significantly except for low density lipoprotein. In the first day after DFPP, the serum level of TG, cholesterol and very low density lipoprotein declined significantly, while the high-density lipoprotein (HDL) as well as apoprotein A1 elevated obviously (*P* < 0.05). TG maintained the downward trend in the following three days and the other parameters kept steady. Linear regression analysis showed that HDL was negatively correlated with the duration of hospitalization among three adjusted models (*P* = 0.043, *P* = 0.029, *P* = 0.025 respectively).

**Conclusion:**

There was distinct fluctuation of the lipid profile upon the burst of sHTGP and the parameters changed significantly in the first day after DFPP. Among these parameters, HDL may serve as a biomarker for disease prognosis in patients with sHTGP.

## Background

Acute pancreatitis (AP) is an inflammatory disease of the pancreas with a worldwide incidence of 13–80 cases per 100,000 per annum [1, 2]. Metabolic syndrome was proved to be closely related to the development and/or severity of AP [3, 4]. As one of the components of metabolic syndrome, severe hypertriglyceridemia (sHTG), defined as a serum triglyceride (TG) level > 11.3 mmol/L, is a common cause of AP [5, 6]. It has been found that the incidence of severe hypertriglyceridemia-induced acute pancreatitis (sHTGP) increased gradually and became the second common etiology of AP in China [7]. Lipoprotein metabolism disturbance has close association with the development and prognosis of sHTGP [8]. For example, the elevated TG level on admission was considered to be a risk factor for poor prognosis of sHTGP [9–11]. However, there was no systemic evaluation of lipid profile changes during sHTGP as so far.

It is generally considered that efficient and rapid TG-lowering therapy is one of the essential management for sHTGP. Some studies suggested TG < 5.6 mmol/L as an ideal lipid-lowering target [12]. TG levels dropped rapidly after fasting in most HTG-induced AP patients while the effect was unsatisfactory in sHTGP patients. Intravenous heparin and insulin or insulin only were two common drug therapies for lowering TG, while the effect was controversial and a currently ongoing study is trying to figure it out [13]. Plasmapheresis is considered to be one of the most effective therapies for lowering TG rapidly in the setting of sHTGP [7]. Double filtration plasmapheresis (DFPP) is a semi-selective apheresis method based on a double filter system, which can remove macromolecules selectively [14]. There is no study reporting the dynamic changes of lipid profile under DFPP in patients with sHTGP by far. This study aimed to characterize dynamic lipid profile changes in sHTGP patients after DFPP, and ascertain the association between these changes and the clinical prognosis.

## Methods

### Patients

This was a retrospective observational study carried out in the Department of Critical Care Medicine of Sir Run Run Shaw Hospital (a university-affiliated hospital in Hangzhou, China). The study included consecutive patients with sHTGP (admission TG > 11.3 mmol/L) who were admitted within 72 h after AP onset. From January 2019 to February 2020, there were 494 patients admitted to the Sir Run Run Shaw Hospital during the period, and the main etiologies were cholelithiasis (47.4%), hypertriglyceridemia (HTG, 39.9%) and alcohol (4.9%) respectively. AP was diagnosed according to the 2012 Atlanta classification [15]. Exclusion criteria included: age < 18 years old, pregnancy, complicated with malignant tumor and incomplete information. The study was approved by the Ethics Committee of Sir Run Run Shaw Hospital (20190215–3). Because of the retrospective characteristic of the study, Informed consents from individuals were waived.

### Intervention

According to the AP treatment procedures of the center, all the sHTGP patients were suggested to receive DFPP within 24 h after admission. DFPP was conducted via femoral double lumen using Plasauto EZ machine machine (Asahi-Kasei, Tokyo, Japan), which was loaded with a blood cell separator column (Plasmaflo, OP-08) and a plasma separator (Cascadeflo, EC-40 W). Heparin was applied for the anticoagulation of the system. Large weight molecules such as VLDL were discarded and clean plasma was returned to circulation system. The frequencies of DFPP sessions were decided by the clinicians in each case.

### Data collection

Baseline characteristics included demographic data, body mass index (BMI), co-morbidities, disease severity, the Acute Physiology and Chronic Health Evaluation II score (APACHE II) and the Sequential Organ Failure Assessment score (SOFA). The lipid profile was collected in each case on admission, consecutive 4 days after DFPP and at discharge. Lipid profile included serum TG, cholesterol (TC), very low density lipoprotein (VLDL), low density lipoprotein (LDL), HDL and Apoprotein A1 (Apo A1). TG, TC, VLDL, LDL, HDL and Apo A1 were measured by homogeneous assays carried out by the Abbott ARCHITECT c16000 Clinical Chemistry Analyser (Abbott Diagnostics, USA). The primary outcome in this study was the hospitalization duration.

### Statistical analysis

For continuous variable with normal distribution, it was presented as mean ± SD and analyzed using *t* test. For continuous variable without normal distribution, it was presented as median (25, 75% interquartile ranges) and analyzed using Mann-Whitney test. Categorical variables were presented as percentages and analyzed with the χ2 test. Propensity score matching was used to minimize the effect of confounding factors which may lead to outcome bias. A one-to-one nearest neighbor matching algorithm was applied using a caliper width of 0.05. The following variables were selected to generate the propensity score: age, sex, BMI, APCHE II, SOFA and initial TG level. Kernel density plots of the p score were applied to examine the propensity score matching degree. Finally, 10 matched pairs were generated and applied to further analyses. After coinciding with all assumptions, multiple linear regression analyses were applied to explore the relationship between parameters of lipid profile and hospitalization duration. Four models were used in the linear regression analyses: model 1 was performed as an adjusted model; model 2 was adjusted for age, sex and BMI; model 3 was adjusted for age, sex BMI and APACHE II score; model 4 was adjusted for age, sex BMI, APACHE II score and co-morbidities. The statistical analysis was performed by SPSS 23.0 and Graphpad Prism 6.0. *P* values < 0.05 were considered statistically significant (two-tailed). In this study, the continuous variables with missing values were less than 20% and consequently the missing values were replaced by the mean or median values.

## Results

### Baseline and clinical characteristics of patients with sHTGP

This study included 47 sHTGP patients and the baseline characteristics are presented in Table 1. The median days from AP onset to admission was 1 and the median serum TG on admission was 42.9 mmol/L (minimum: 12.8 mmol/L, maximum: 190.9 mmol/L). According to the 2012 Atlanta classification, 24% patients met the diagnostic criteria of severe acute pancreatitis (SAP). 48.9% patients had recurrent AP (recurrence times: 2 [1, 3]) and 36.1% patients were complicated with diabetes. According to whether they had AP recurrence, sHTGP patients were divided into two groups: the non-recurrence and recurrence group. There were no significant differences between the two groups in baseline and clinical characteristics, except diabetes. The percentage of diabetes was significantly higher in the recurrence group, suggesting diabetes may be a risk factor for sHTGP recurrence (52% vs 21%, *P* = 0.025).

**Table 1:** Baseline and clinical characteristics of the sHTGP patients

Variables	All patients (*n* = 47)	non-recurrence (*n* = 24)	recurrence (*n* = 23)	*P*
Age, mean ± SD, y	37.7 ± 9.6	37.2 ± 7.9	38.2 ± 11.3	0.713
Male, n (%)	32 (68)	14 (58)	18 (78)	0.143
BMI, mean ± SD, kg/m^2^	26.9 ± 3.4	25.3 ± 4.0	27.7 ± 2.7	0.338
Disease severity, n (%)				0.897
Mild	27 (57.4)	14 (58.4)	13 (56.5)	
Moderate	9 (19.2)	5 (20.8)	4 (17.4)	
Severe	11 (23.4)	5 (20.8)	6 (26.1)	
APACHE II, median (ranges)	8.0 (6.0, 13.0)	7.5 (6.0, 14.3)	8.0 (5.5, 14.3)	0.927
SOFA, median (ranges)	2.0 (1.0, 3.0)	2.0 (1.0, 3.0)	2.0 (1.0, 3.0)	0.981
Co-morbidities, n (%)				
Hypertension	8 (17.0)	4 (16.7)	4 (17.4)	0.947
Diabetes	17 (36.1)	5 (20.8)	12 (52.2)	0.025
ICU days, median (ranges)	3 (2, 7)	5 (1, 7)	3 (2, 6)	0.914
Hospital days, median (ranges)	12 (8, 16)	14 (9, 16)	10 (7, 15)	0.105

### Dynamic changes in parameters of lipid profile for sHTGP patients

**Fig. 1:**
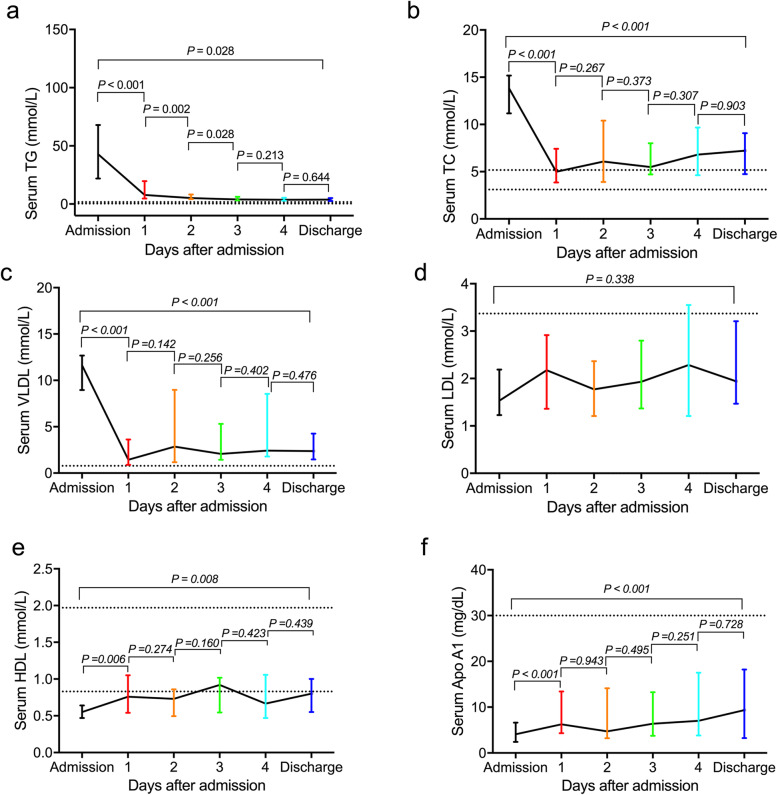
Dynamic changes of lipid profile before and after DFPP during sHTGP. Data are presented as medians, with I bars indicating interquartile ranges. The dotted lines represent the reference standard of these parameters in the center. T-test was used to compare the difference between adjacent two sets of data

All the sHTGP patients received DFPP within 24 h after admission. No complications had been found during DFPP treatment. Serum TG level dropped by an average of 71.2% in the first day after DFPP, and the level in 36.2% patients fell below the safety limit (5.6 mmol/L). The level of TG continued to decrease significantly in next 3 days and stalled by day 4 (Fig. 1a). At discharge, there were 78.7% patients whose TG level was under the safety limit. The levels of TC and VLDL were higher than the normal range in all the sHTGP patients on admission. It was found that the levels of TC and VLDL decreased significantly after DFPP in day 1 and then kept stable until discharge (Fig. 1 b-c). The HDL level was lower than normal range on admission among 89% patients and the level increased obviously after DFPP in day 1 (Fig. 1e). Apo A1 is the major component of HDL and its variation exhibited the same tendency with HDL (Fig. 1f). Interestingly, serum LDL maintained normal level in 94% patients and kept stable after DFPP (Fig. 1d). All the patients had resumed oral diet at discharge and their lipid profile showed distinct difference from the admission data. The levels of TG, TC and VLDL at discharge were significantly lower than admission, while the levels of HDL and Apo A1 at discharge were significantly higher than admission (Fig. 1).

### Association between TG-lowering efficiency and clinical results

It is widely accepted that the control of TG level below 5.67 mmol/L appear to hold prognostic value. To figure out the effect of lipid-lowering efficiency on patients’ prognosis, patients were divided into two groups according to the TG level after DFPP on the first day: target group (TG < 5.67 mmol/L) and non-target group (TG ≥ 5.67 mmol/L). A propensity score matching was performed to eliminate the influence of initial TG level on prognosis and finally 10 pairs were matched (Table 2). The ICU days for non-target group were longer than target group with no statistical difference (*P* = 0.089). Compared to non-target group, the target group had significantly shorter hospital days (*P* = 0.035).

**Table 2:** Baseline and outcome of matched sHTGP patients

Variables	Non-target (*n* = 10)	Target (n = 10)	*P*
Age, mean ± SD, y	38.6 ± 7.1	39.0 ± 9.4	0.930
Male, n (%)	9 (90)	6 (60)	0.121
BMI, mean ± SD, kg/m^2^	27.5 ± 3.7	26.7 ± 3.6	0.624
APACHE II, median (ranges)	7 (4, 12)	6 (4, 11)	0.824
SOFA, median (ranges)	1.5 (0.3, 2.8)	2 (1.0, 2.3)	0.783
Serum TG, mean ± SD, mmol/L	31.7 ± 13.0	32.8 ± 17.1	0.870
ICU days, median (ranges)	6.5 (3.3, 7.5)	1.5 (1.0, 4.3)	0.089
Hospital days, median (ranges)	14.5 (13.0, 16.0)	8.0 (6.0, 15.3)	0.035

### Associations between lipid profile and clinical results

To explore the relationship between the other lipid profile parameters and clinical results, linear regression analyses were performed based on four models. The results showed that the decrease in serum HDL on admission was significantly associated with longer hospitalization in three models (*P* = 0.043, *P* = 0.029, *P* = 0.025 for model 1, 3, 4 respectively). APACHE II had a significant association with hospitalization in model 2, 3 and 4. However, the length of hospitalization was not significantly associated with the other parameters on admission (Table 3). The relationship between lipid profile and length of hospitalization after DFPP was analyzed and no significance was found (data not shown).

**Table 3:** Linear regression analyses for parameters of lipid profile with hospital days

Parameters	model	β	95%CI		*P*
			lower	upper	
TC	1	− 0.039	− 0.617	0.475	0.795
	2	−0.004	− 0.622	0.606	0.979
	3	−0.051	−0.722	0.535	0.764
	4	−0.101	−0.833	0.459	0.561
VLDL	1	0.037	−0.482	0.617	0.805
	2	0.069	−0.476	0.728	0.674
	3	0.002	−0.608	0.616	0.990
	4	−0.041	−0.705	0.553	0.808
LDL	1	−0.038	−2.195	1.706	0.802
	2	−0.038	−2.291	1.790	0.806
	3	−0.075	− 2.663	1.649	0.637
	4	−0.078	−2.694	1.647	0.628
HDL	1	−0.297	−13.783	−0.236	**0.043**
	2	−0.314	−14.939	0.117	0.053
	3	−0.346	−15.370	−0.882	**0.029**
	4	−0.356	−15.634	−1.128	**0.025**
Apo A1	1	−0.058	−0.683	0.463	0.700
	2	−0.017	−0.695	0.631	0.923
	3	−0.059	−0.768	0.543	0.730
	4	−0.087	−0.851	0.519	0.626

## Discussion

This study investigated the variation of lipid profile after DFPP in a cohort of sHTGP patients. The average age of this cohort was 37.7, which is younger than other countries cohorts. It has been observed that the age of HTG-associated AP patients tended to be lower recent years in China, which may be attributed to the change of lifestyle [6]. It is widely considered that the increase in serum TG levels has a positive correlation with disease severity and AP recurrence [12]. 23.4% sHTGP patients in this study developed SAP and the proportion was only 9.6% in whole HTG-induced pancreatitis cohort as reported previously [16]. In addition, the average BMI of the cohort in this study was 26.9, and Dobszai et al. demonstrated that a BMI above 25 increases the risk of SAP [17]. It was previously reported that the recurrence rate of HTG-induced pancreatitis was about 25–30%, while the rate was as high as 50% in this study [16, 18]. A longitudinal cohort study on HTG-induced pancreatitis found that even mild elevation in TG level was associated with increased risk of AP recurrence compared to patients who returned normal TG (RR 5.47 [1.80, 16.65]) [19]. It is generally considered that patients with HTG are often complicated with metabolic abnormalities such as diabetes [8, 20]. 36.1% sHTGP patients in this cohort were complicated with diabetes and the proportion was higher in the recurrence group. The presence of diabetes was considered to be an independent risk factor for AP recurrence [21]. The results were in accordance with previous studies. Aune et al. reviewed the link between diabetes and AP, and the results concluded that the relative risk for developing AP was 1.74 in patients with diabetes compared with patients without diabetes [20].

This study found that the lipid profile of sHTGP changed obviously after DFPP in the first day and the level of parameters maintained stability except for TG. All the patients resumed oral feeding at discharge and the level of parameters at this point may serve as the normal reference. Interestingly, the levels of lipid profile at disease onset exhibited significant difference from the normal level of patients, indicating the burst of sHTGP may be concerned with an acute and dramatic fluctuation of the lipid metabolism. The results conform to the prevalent speculations for the pathogenesis of sHTGP [10].

Reduction of TG levels sufficiently is thought to be critical to the effective management of HTG-induced pancreatitis [22]. However, the pharmacologic dyslipidemia therapies such as insulin and heparin were found to be insufficient to lowering TG rapidly [23]. DFPP has been used to lower TG levels rapidly in sHTGP patients for decades. According to a systemic review published in 2017, apheresis can reduce the initial serum TG (mean: 42.0 mmol/L) by 72%, and the result of this study showed similar efficiency (pretreatment TG: 42.68 mmol/L by 71.2%). There were few studies exploring the effect of DFPP on sHTGP management. Chang et al. investigated the effectiveness of DFPP in 12 sHTGP patients and found that DFPP had shorten the hospitalization duration and minimized the recurrence rate [24]. This study showed similar results.

Lipids and lipoproteins undergo changes during inflammatory diseases and may be used as potential biomarkers (especially HDL) [25]. It was observed that critically ill patients showed low concentrations of HDL and Apo A1 upon ICU admission which were correlated with increased disease severity, ICU duration and mortality [26, 27]. Similar to findings in previous studies, this study found that decreased HDL appears to be indicative of the extension of sHTGP hospitalization [28, 29]. HDL is the complex of lipoprotein species which contain approximately 25% of the cholesterol and < 5% of the TG in human blood, playing a major role in TG transport and removal [30]. It is generally considered that HDL has potent anti-inflammatory properties which may be important for protection against AP and other inflammatory disease through modulating macrophages reprogramming [29, 31]. Lower levels of HDL were elucidated to be associated with increased cardiovascular events and poor outcomes [32, 33]. Bugdaci et al. reported that the levels of HDL were negatively associated with the Ranson score of AP [28]. Khan et al. found that levels of serum HDL was significantly lower in SAP patients and was associated with longer hospitalization [29].

### Strength and limitations

This is the first study which systematically evaluated the influence of DFPP on the lipid profile in sHTGP patients. However, there were several limitations of this study. First, this was a retrospective analysis and the study included relatively small number of patients due to the single center design. Second, this study lacks the control group for comparison with the DFPP group. In the real-world practice, all the sHTGP patients received DFPP on admission in this center and consequently the study lacks untreated sHTGP cohort. Further large scale and multicenter studies are needed.

## Conclusions

In summary, there was distinct fluctuation of the lipid profile upon the burst of sHTGP and the parameters changed significantly in the first day after DFPP. Among these parameters, HDL may serve as a biomarker of disease prognosis in patients with sHTGP. The results of this study improve the perceive of lipid profile in the clinical course of sHTGP and indicate the necessity of lipid monitoring during disease progression.

## Data Availability

All data generated and analyzed in this study are included in this published article. The datasets are available from the corresponding author on reasonable request.

## Abbreviations

AP: Acute pancreatitis
APACHE II: Acute Physiology and Chronic Health Evaluation score II
Apo A1: Apoprotein A1
BMI: Body mass index
DFPP: Double filtration plasmapheresis
HDL: High density lipoprotein
ICU: Intensive care unit
LDL: Low density lipoprotein
sHTG: Severe hypertriglyceridemia
sHTGP: Severe hypertriglyceridemia-induced acute pancreatitis
SAP: Severe acute pancreatitis
SOFA: Sequential organ failure assessment score
TC: Total cholesterol
VLDL: Very low density lipoprotein
